# P-481. Commensals in Early-Onset Sepsis Blood Cultures: A Common Driver of Increased Antibiotic Days and Length of Stay

**DOI:** 10.1093/ofid/ofaf695.696

**Published:** 2026-01-11

**Authors:** Erica Prochaska, Nora Elhaissouni, Elizabeth Colantuoni, Veeral Tolia, Daniel Benjamin, Sagori Mukhopadyay, Aaron Milstone

**Affiliations:** Johns Hopkins University, Columbia, MD; Johns Hopkins University School of Medicine, Baltimore City, Maryland; Bloomberg School of Public Health, Johns Hopkins University, Baltimore City, Maryland; Pediatrix Medical Group, Dallas, Texas; Duke University School of Medicine, Durham, North Carolina; CHOP/Penn, Philadelphia, Pennsylvania; Johns Hopkins University, Columbia, MD

## Abstract

**Background:**

Contaminated blood cultures are a driver of healthcare and antibiotic overuse. The burden of neonatal contaminated blood cultures is unknown. Within a retrospective cohort of neonates born ≥37 weeks gestational age who had a blood culture obtained in the first 3 days after birth with no central line in place, our objectives were to 1) estimate the proportion of blood cultures that grew commensals, and 2) to compare the length of stay and antibiotic use among neonates with a commensal versus negative blood culture.

**Figure 1:**
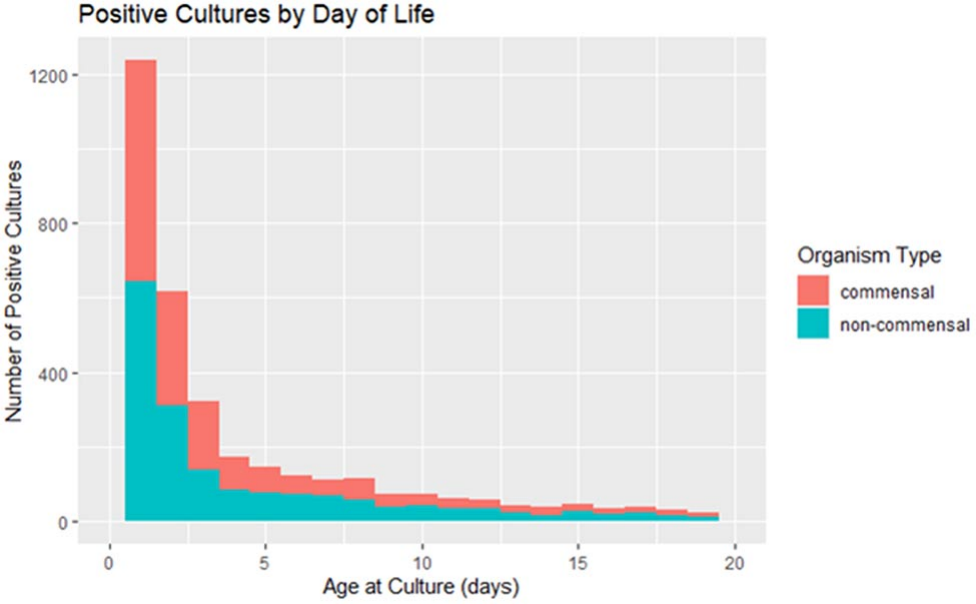
Stacked bar graph of positive neonatal blood cultures, stratified by commensal and non-commensal organisms.

**Figure 2:**
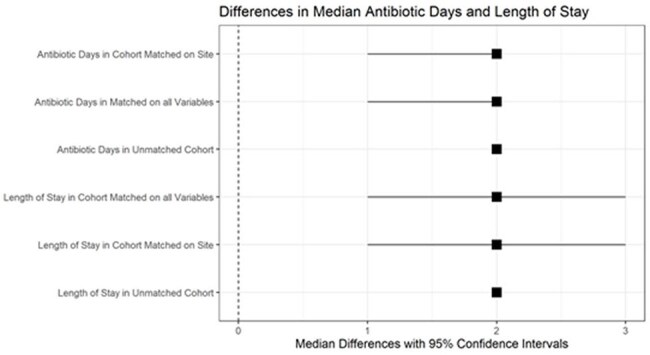
Unadjusted and adjusted differences in median antibiotic days and length of stay among infants with a commensal versus negative blood culture. Confidence intervals calculated using a bootstrap with resamples taken within NICU.

**Methods:**

The cohort was extracted from infants admitted to a 315 Pediatrix NICUs between 2016-2021. First, we estimated the total proportion of commensals out of all positive blood cultures in the cohort. For the outcomes analysis, we excluded infants who were transferred out of the NICU and who had a non-commensal blood culture in the first 14 days after birth. We then compared infants with a single positive commensal blood culture (cases) and infants with a negative blood culture (controls) to estimate the difference in median length of stay and antibiotic days using 3 approaches: 1) unadjusted comparison 2) matching on NICU, and 3) matching on duration of rupture of membranes, maternal group B streptococcus status, intrapartum antibiotic use and NICU. Confidence intervals for approaches 2 and 3 were derived using a bootstrap (1,000 resamples of infants taken within NICU).

**Results:**

There were 2,179 positive cultures in the cohort, 1,082 (50%) grew a commensal organism (Figure 1). The proportion of positive blood cultures that were commensals varied across the NICUs (median of 50%, interquartile range: 31% to 73%). In our outcomes analysis, there were 115,095 infants, 767 who had a had a commensal blood culture within the first 3 days after birth. Of these cases, 337 (44%) were treated with ≥5 days of continuous antibiotics. Cases had a median excess of 2 antibiotic and admission days as compared to controls in the matched and unmatched analyses (Figure 2).

**Conclusion:**

Commensals grew in 50% of positive blood cultures collected for early-onset sepsis in a large NICU cohort. These events were associated with increased antibiotic use and length of stay. Improving infant blood culture collection quality is needed to reduce false-positive tests and antibiotic exposure in this vulnerable population.

**Disclosures:**

Daniel Benjamin, Jr., MD, PhD, MPH, AbbVie, Inc.: Advisor/Consultant|PPD, Inc.: Advisor/Consultant|Syneos Health: Advisor/Consultant

